# Cloud computing applications for biomedical science: A perspective

**DOI:** 10.1371/journal.pcbi.1006144

**Published:** 2018-06-14

**Authors:** Vivek Navale, Philip E. Bourne

**Affiliations:** 1 Center for Information Technology, National Institutes of Health, Bethesda, Maryland, United States of America; 2 Department of Biomedical Engineering, University of Virginia, Charlottesville, Virginia, United States of America; Genome Quebec, CANADA

## Abstract

Biomedical research has become a digital data–intensive endeavor, relying on secure and scalable computing, storage, and network infrastructure, which has traditionally been purchased, supported, and maintained locally. For certain types of biomedical applications, cloud computing has emerged as an alternative to locally maintained traditional computing approaches. Cloud computing offers users pay-as-you-go access to services such as hardware infrastructure, platforms, and software for solving common biomedical computational problems. Cloud computing services offer secure on-demand storage and analysis and are differentiated from traditional high-performance computing by their rapid availability and scalability of services. As such, cloud services are engineered to address big data problems and enhance the likelihood of data and analytics sharing, reproducibility, and reuse. Here, we provide an introductory perspective on cloud computing to help the reader determine its value to their own research.

## Introduction

Progress in biomedical research is increasingly driven by insight gained through the analysis and interpretation of large and complex data sets. As the ability to generate and test hypotheses using high-throughput technologies has become technically more feasible and even commonplace, the challenge of gaining useful knowledge has shifted from the wet bench to include the computer. Desktop computers, high-performance workstations, and high-performance computing systems (HPC clusters) are currently the workhorses of the biomedical digital data research endeavor. Recently, however, cloud computing, enabled by the broad adoption and increasing capabilities of the internet and driven by market need, has emerged as a powerful, flexible, and scalable approach to disparate computational and data–intensive problems. The National Institute of Standards and Technology (NIST) states the following:

Cloud computing is a model for enabling convenient, on-demand network access to a shared pool of configurable computing resources (e.g., networks, servers, storage applications and services) that can be rapidly provisioned and released with minimal management effort or service provider interaction.

NIST categorizes clouds as one of 4 types: public, private, community, and hybrid. In a public cloud, the infrastructure exists on cloud provider premises and is managed by the cloud provider, whereas in a private cloud, the infrastructure can exist on or off the premises of the cloud provider but is managed by the private organization. Examples of public clouds include Amazon Web Services (AWS), Google Cloud Platform (GCP), and Microsoft Azure. A community cloud is a collaborative effort where infrastructure is shared between several organizations—a specific community—that have common requirements for security and compliance. For example, the Federal Risk and Authorization Management Program (FedRAMP) is a United States government-wide program that provides a standardized approach to security assessment, authorization, and continuous monitoring of information technology (IT) infrastructure [1]. The AWS GovCloud is an example of a FedRAMP-accredited resource that operates as a community cloud that addresses US government community needs. The JetStream Cloud [2] serves as a community cloud serving the scientific community. A hybrid cloud is a composition of 2 or more distinct cloud infrastructures—private, community, public—that remain unique entities but are bound together in a way that enables portability of data and software applications [3].

Cloud types discussed above can use one or more cloud services—Software as a Service (SaaS), Platform as a Service (PaaS), and Infrastructure as a Service (IaaS). SaaS enables the consumer to use the cloud provider’s applications (e.g., Google Docs) that are running on a cloud provider’s infrastructure, whereas PaaS enables consumers to create or acquire applications and tools and to deploy them on the cloud provider’s infrastructure. IaaS enables a consumer to provision processing, storage, networks, and other fundamental computing resources. Most public cloud providers like AWS, GCP, and Microsoft Azure provide IaaS, PaaS, and SaaS, and the customer can select the best applicable solution for their individual needs.

Cloud adoption, regardless of type, has varied in industry because of different levels of security and other features required for operation. Previously, both public and private clouds have been used more in unregulated industries and to a lesser extent in regulated industries, but this is changing [4]. Federally funded scientific data sets are being made available in public clouds [5]. For example, Human Microbiome Project (HMP) data, funded by the National Institutes of Health (NIH), is available on AWS simple storage service (S3) [6], and more biomedical data sets are becoming available in the cloud. Research investigators can now request permission from NIH to transfer controlled-access genomic and associated phenotypic data obtained from NIH-designated data repositories to public or private cloud systems for data storage and analysis [7]. Subject to appropriate access controls on human subjects’ data, the NIH is committed to making public access to digital data a standard for all NIH-funded research [8].

Advances across the biological scales, from sequencing instruments to health monitoring devices, image collections to the expansion of electronic health record (EHR) platforms, will see a reduced cost in the acquisition of data. Estimates indicate that in 2016, the NIH alone was supporting 650 petabytes (PB) of data at various institutional repositories. Both volume and complexity of biomedical data will significantly increase in coming years, bringing challenges for storage, management, and preservation, suggesting an increased usage of cloud computing. Biomedical research can benefit from the growing number of cloud-based big data tools and platforms being developed and refined for other industries.

## Adopting cloud for biomedical work

Consider examples of how clouds and cloud services have been deployed in biomedical work (Table 1). In genomics alone, usage ranges from single applications to complete virtual machines with multiple applications. Additional information on cloud resources in bioinformatics has been provided previously [9].

**Table 1:** Examples of cloud types, service models, workflows, and platforms for biomedical applications.

Biomedical Use	Cloud Type	Cloud Service Models	Cloud Provider Examples	Additional Notes
**Individual Tools**				
Sequence alignment	Public cloud	IaaS	AWS, Azure, Google	BLAST
Long-sequence mapping	Public cloud	IaaS	AWS	CloudAligner, Elastic MapReduce
Short-sequence mapping	Public cloud	IaaS	AWS	CloudBurst
High-throughput sequencing analysis	Public cloud	IaaS	AWS	Eoulsan package, Elastic MapReduce
Sequence alignment and genotyping	Public cloud	IaaS	AWS	Crossbow, Elastic MapReduce
**Workflows and Platforms**				
NGS and data analysis	Public cloud	IaaS	AWS	Galaxy, open source applications
NGS Analysis	Private cloud	PaaS	Bionimbus Protected Data cloud	OpenStack, software to build cloud platforms
NGS for clinical diagnostic work	Public cloud	PaaS	AWS CloudMan	Cloud Biolinux, Cloud BioCentral
Mutation pattern study in thousands of whole genome sequences	Hybrid cloud	IaaS	AWS EC2 S3	University resources combined with public cloud
Large scale data analysis (TCGA)	Public cloud	PaaS	Google Elastic Compute	Broad Institute FireCloud
Large scale data analysis (TCGA)	Public cloud	PaaS	GCP	Institute for Systems Biology
Large scale data analysis (TCGA)	Public Cloud	PaaS, SaaS	AWS	Seven Bridges cancer genomics cloud interfaced with AWS and GCP
Genomics data analysis	Public cloud	PaaS	AWS	Knowledge Engine Data mining and machine learning
Large scale sequencing, data analysis, and integration of phenotypic and clinical data	Public cloud	PaaS, SaaS	AWS, Microsoft Azure	DNAnexus Deep Variant informatics tool
Workflow applications for genomics	Public cloud	PaaS, SaaS	Google cloud platform	DNAstack Selection of highly curated data pipelines by using Workflow App
**Healthcare**				
Real-time ECG monitoring	Hybrid cloud	IaaS	AWS EC2	Combined use of on-site resources with public cloud
Telemedicine service 12-lead ECG	Public cloud	PaaS	Microsoft Azure	Deployment of secure ECG applications, visualization and data management services with cloud-based database
Diagnostic image storage and retrieval	Public cloud	PaaS	AWS, Microsoft Azure, Google Apps Engine	Hosting of Picture Archive Communication System core modules to set up medical data repositories
**General Purpose Tools**				
Automated microbial sequence analysis	Public cloud	IaaS	AWS EC2	cloVR
High-performance bioinformatics computing	Public cloud	IaaS	AWS	Cloud Biolinux
Biomedical big data	Public cloud	PaaS	AWS, Azure, Google, IBM	Hadoop, MapReduce, BigQuery, Redshift

Abbreviations: NGS, Next Generation Sequencing; AWS, Amazon Web Services; EC2, Elastic Compute Cloud; S3, Simple Storage Service; TCGA, The Cancer Genome Atlas; GCP, Google Cloud Platform; IaaS, Infrastructure as a Service; PaaS, Platform as a Service; SaaS, Software as a Service

### Individual tools

BLAST [10] is one of the most frequently used tools in bioinformatics research. A BLAST server image can be hosted on AWS, Azure, and GCP public clouds to allow users to run stand-alone searches with BLAST. Users can also submit searches using BLAST through the National Center for Biotechnology Information (NCBI) application programming interface (API) to run on AWS and Google Compute Engine [11]. Additionally, the Microsoft Azure platform can be leveraged to execute large BLAST sequence matching tasks within reasonable time limits. Azure enables users to download sequence databases from NCBI, run different BLAST programs on a specified input against the sequence databases, and generate visualizations from the results for easy analysis. Azure also provides a way to create a web-based user interface for scheduling and tracking the BLAST match tasks, visualizing results, managing users, and performing basic tasks [12].

CloudAligner is a fast and full-featured MapReduce-based tool for sequence mapping, designed to be able to deal with long sequences [13], whereas CloudBurst [14] can provide highly sensitive short read mapping with MapReduce. High-throughput sequencing analyses can be carried out by the Eoulsan package integrated in a cloud IaaS environment [15]. For whole genome resequencing analysis, Crossbow [16] is a scalable software pipeline. Crossbow combines Bowtie, an ultrafast and memory efficient short read aligner, and SoapSNP, a genotyper, in an automatic parallel pipeline that can run in the cloud.

### Workflows and platforms

Integration of genotype, phenotype, and clinical data is important for biomedical research. Biomedical platforms can provide an environment for establishing an end-to-end pipeline for data acquisition, storage, and analysis.

Galaxy, an open source, web-based platform, is used for data–intensive biomedical research [17]. For large scale data analysis, Galaxy can be hosted in cloud IaaS (see tutorial [18]). Reliable and highly scalable cloud-based workflow systems for next-generation sequencing analyses has been achieved by integrating the Galaxy workflow system with Globus Provision [19].

The Bionimbus Protected Data Cloud (BPDC) is a private cloud-based infrastructure for managing, analyzing, and sharing large amounts of genomics and phenotypic data in a secure environment, which was used for gene fusion studies [20]. BPDC is primarily based on OpenStack, open source software that provides tools to build cloud platforms [21], with a service portal for a single point of entry and a single sign-on for various available BPDC resources. Using BPDC, data analysis for the acute myeloid leukemia (AML) resequencing project was rapidly performed to identify somatic variants expressed in adverse-risk primary AML samples [22].

Scalable and robust infrastructure for Next Generation Sequencing (NGS) analysis is needed for diagnostic work in clinical laboratories. CloudMan is available on the AWS cloud infrastructure [23]. It has been used as a platform for distributing tools, data, and analysis results. Improvements in using CloudMan for genetic variant analysis has been carried out by reducing storage costs for clinical analysis work [24].

As part of the Pan Cancer Analysis of Whole Genomes (PCAWG), common patterns of mutation in over 2,800 cancer whole genome sequences were studied, which required significant scientific computing resources to investigate the role of the noncoding parts of the cancer genome and for comparing genomes of tumor and normal cells [25]. The PCAWG data coordinating center currently lists collaborative agreements with cloud provider AWS and the Cancer Collaboratory [26], an academic compute cloud resource maintained by the Ontario Institute for Cancer Research and hosted at the Compute Canada facility.

Multiple academic resources were used to complete analysis of 1,827 samples taking over 6 months. This was supplemented by the use of cloud resources, where 500 samples were analyzed by AWS in 6 weeks [27]. This showed that public cloud resources can be rapidly provisioned to quickly scale up a project if increased compute resources are needed. In this instance, AWS S3 data storage was used to scale from 600 terabytes to multiple PBs. Raw reads, genome alignments, metadata, and curated data can also be incrementally uploaded to AWS S3 for rapid access by the cancer research community. Data search and access tools are also available for other researchers to use or reuse. Sequence read-level data and germline data are maintained at the controlled tier of the cloud, and access to read data requires preapproval from the International Cancer Genome Consortium (ICGC) data access compliance office.

The National Cancer Institute (NCI) has funded 3 cloud pilots to provide genomic analysis, computational support, and access capabilities to the Cancer Genome Atlas (TCGA) data [28]. The objective of the pilots was to develop a scalable platform to facilitate research collaboration and data reuse. All 3 cloud pilots have received authoritative and harmonized reference data sets from the cancer Genomic Data Commons (GDC) [29] that have been analyzed using a common set of workflows against a reference genome (e.g., GRCh38). The Broad Institute pilot developed FireCloud [30] using the elastic compute capacity of Google Cloud for large-scale data analysis, curation, storage, and data sharing. Users can also upload their own analysis methods and data to workspaces and/or use Broad Institute’s best practice tools and pipelines on preloaded data. FireCloud uses the Workflow Description Language (WDL) to enable users to run scalable, reproducible workflows [31].

The Institute for Systems Biology (ISB) pilot leverages several services on the GCP. Researchers can use web-based software applications to interactively define and compare cohorts, examine the underlying molecular data for specific genes or pathways of interest, share insights with collaborators, and apply their individual software scripts and programs to various data sets [32].

The ISB Cancer Genome Cloud (CGC) has loaded processed data and TCGA project metadata into the BigQuery managed database service, enabling easy data mining and data warehouse approaches to be used on large-scale genomics data. The Seven Bridges Genomics (SBG) CGC offers both genomics SaaS and PaaS and uses AWS [33]. The platform also enables researchers to collaborate on the analysis of large cancer genomics data sets in a secure, reproducible, and scalable manner. SGB CGC implements Common Work-Flow language [34] to facilitate developers, analysts, and biologists to deploy, customize, and run reproducible analysis methods. Users may choose from over 200 tools and workflows covering many aspects of genomics data processing to apply to TCGA data or their own data sets.

Efforts are underway by the NIH Center for Excellence in Big Data Computing at the University of Illinois, Urbana-Champaign to construct a Knowledge Engine for Genomics (KnowEnG). The KnowEnG system is deployed on a public cloud infrastructure—currently AWS—to enable biomedical scientists to access data-mining, network-mining, and machine-learning algorithms that can aid in extracting knowledge from genomics data [35]. A massive knowledge base of community data sets called the Knowledge Network is at the heart of the KnowEnG system, and data sets, even those in spreadsheets, can be brought to KnowEnG for analysis.

Commercial (AWS, Microsoft Azure) cloud-based platforms (e.g., DNAnexus) enables analyses of massive amounts of sequencing data integrated with phenotypic or clinical information [36]. Also, the application of deep learning-based data analysis tools (e.g., Deep Variant) in conjunction with DNAnexus have been used to call genetic variants from next-generation sequencing data [37]. Other bioinformatics platforms (e.g., DNAstack) use the GCP for providing processing capability for over a quarter of a million whole human genome sequences per year [38].

### Healthcare

Cloud computing applications in healthcare include telemedicine/teleconsultation, medical imaging, public health, patient self-management, hospital management and information systems, therapy, and secondary use of data.

Real-time health monitoring for patients with chronic conditions who reside at considerable distances from their health service providers have difficulty in having their health conditions monitored. One poignant example are patients who suffer from cardiac arrhythmias requiring continuous episode detection and monitoring. Wearable sensors can be used for real-time electrocardiogram (ECG) monitoring, arrhythmia episode detection, and classification. Using AWS EC2, mobile computing technologies were integrated, and ECG monitoring capabilities were demonstrated for recording, analyzing, and visually displaying data from patients at remote locations. In addition, software tools that monitored and analyzed ECG data were made available via cloud SaaS for public use [39]. Also, the Microsoft Azure platform has been implemented for a 12-lead ECG telemedicine service [40]. For storage and retrieval of medical images, deployment of Picture Archive and Communication System modules were deployed in a public cloud [41]. A review of publications on cloud computing in healthcare has pointed out that many healthcare-related publications have used the term “cloud” synonymously with “virtual machines” or “web-based tools”, not consistent with characteristics that define cloud computing, models, and services [42]. Several commercial vendors are interacting with hospitals and healthcare providers to establish healthcare services through cloud computing options.

### General purpose tools

CloVR is a virtual machine that emulates a computer system, with preinstalled libraries and packages for biological data analysis [43]. Similarly, Cloud BioLinux is a publicly available resource with virtual machine images and provides over 100 software packages for high-performance bioinformatics computing [44]. Both (CloVR and BioLinux) virtual machine images are available for use within a cloud IaaS environment.

Cloud adoption can also include managed services that are designed for general Big Data problems. For example, each of the major public cloud providers offer a suite of services for machine learning and artificial intelligence, some of which are pretrained to solve common problems, (e.g., text-to-speech). Database systems such as Google BigQuery [45] and Amazon Redshift [46] combine the scalable and elastic nature of the cloud with tuned software and hardware solutions to deliver database capabilities and performance not easily achieved otherwise. For large, complex biomedical data sets, such databases can reduce management costs, ease database adoption, and facilitate analysis. Several big data applications used in biomedical research, such as the Apache Hadoop software library, are cloud based [47].

## Developing a cloud-based digital ecosystem for biomedical research

The examples introduced above, some ongoing for several years, illustrate a departure from the traditional approach to biomedical computing. The traditional approach has been to download data to local computing systems from public sites and then perform data processing, analysis, and visualization locally. The download time, cost, and redundancy involved for enhancing local computing capabilities to meet data intensive biomedical research needs (e.g., in sequencing and imaging) makes this approach worthy of re-evaluation.

Large-scale projects, like PCAWG introduced above, have shown the advantage of using resources, both local and public cloud, from various collaborating institutions. For institutions with established on-premises infrastructure (e.g., high-speed network infrastructure, secure data repositories), developing a cloud-based digital ecosystem with options to leverage any of the cloud types (public, hybrid) can be advantageous. Moreover, developing and utilizing a cloud-based ecosystem increases the likelihood of open science.

To promote knowledge discovery and innovation, open data and analytics should be findable, accessible, interoperable, and reusable (FAIR). The FAIR principles serve as a guide for data producers, stewards, and disseminators for enhancing reusability of data, inclusive of data algorithms, tools, and workflows that are essential for good data lifecycle management [48]. A biomedical data ecosystem should have capabilities for indexing of data, metadata, software, and other digital objects—a hallmark of the NIH Big Data to Knowledge (BD2K) initiative [49].

Being FAIR is facilitated by an emerging paradigm for running complex, interrelated sets of software tools, like those used in genomics data processing, and involves packaging software using Linux container technologies, such as Docker, and then orchestrating “pipelines” using domain-specific workflow languages such as WDL and Common Workflow Language [34]. Cloud providers also provide batch processing (e.g., AWS Batch) capabilities that automatically provision the optimal quantity and type of compute resources based on the volume and specific resource requirements of the batch jobs submitted, thereby significantly facilitating analysis at scale.

**Fig 1:**
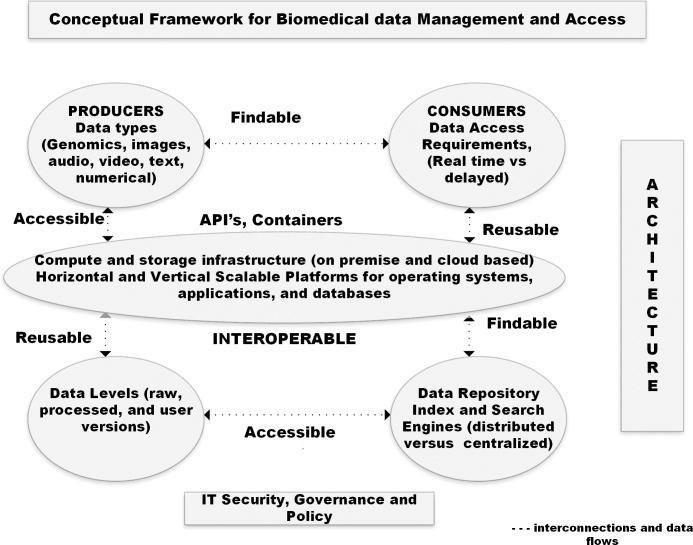
Conceptual cloud-based platform with different data types that flow between producers and consumers requiring variable data level needs.

In Fig 1, we illustrate integration of data producers, consumers, and repositories via a cloud-based platform for supporting the FAIR principles.

The core of a cloud-based platform should support the notion of a commons—a shared ecosystem maximizing access and reuse of biomedical research data and methods.

A cloud-based commons ecosystem can collocate computing capacity, storage resources, database, with informatics tools and applications for analyzing and sharing data by the research community. For multiple commons to interoperate with each other there are 6 essential requirements—permanent digital IDs, permanent metadata, APIs, data portability, data peering, and pay for compute [50].

Other features of the ecosystem include indexing and search capabilities similar to DataMed [51] and a metalearning framework for ranking and selection of the best predictive algorithms [52]. Many of the bioinformatics software tools that we have discussed in the previous section have been successfully deployed in cloud environments and can be adapted to the commons ecosystem, including Apache Spark, a successor to Apache Hadoop and MapReduce for data analysis of Next Generation Sequencing Data [53]. In addition, the data transfer and sharing component of the cloud-based commons ecosystem can include features discussed for the Globus Research Data Management Platform [54]. We also envision cloud-based commons to be supported by techniques and methods that use a semantic data–driven discovery platform designed to continuously grow knowledge from a multitude of genomic, molecular, and clinical data [55].

Security is an integral part of a cloud commons architecture along with data policy, governance, and a business case for sustaining a biomedical digital ecosystem. For initial security controls assessment, guidance documents such as Federal Information Security Management Act (FISMA), NIST-800-53, and Federal Information Processing Standards (FIPS) can provide tools for an organizational assessment of risk and for validation purposes [56,57,58]. Security in public cloud services is a shared responsibility, with the cloud provider providing security services and the end user maintaining responsibility for data and software that leverage those services. A wide range of issues involving ethical, legal, policy, and technical boundaries influence data privacy, all of which are dependent on the type of data being processed and supported [59].

A regular training program for data users of the cloud, especially for handling sensitive data (e.g., personally identifiable information) is important. The training should include methods for securing data that is moved to the cloud, and controlling access to the cloud resources, including virtual machines, containers, and cloud services that are involved for data life cycle management. Protecting access keys, using multifactor authentication, creating identity and access management user lists with controlled permissions, following the principle of least privilege—configured to perform actions that are needed for the users—are some of the recommended practices that can minimize security vulnerabilities that could arise from inexperienced cloud users and/or from malicious external entities [54].

Assessing risk is key to reliably determining the required level of protection needed for data in the cloud. A structured questionnaire approach developed as a Cloud Service Evaluation Model (CSEM) can be used to ascertain risks prior to migration of data to the cloud [60]. Based on the results of risk assessment, a suitable cloud deployment model can be chosen to ensure compliance with internal policies, legal, and regulatory requirements, which, externally, differ in different parts of the world, potentially impacting the ubiquitous nature of cloud resources.

Striving towards open biomedical data has motivated an interest in improving data access while maintaining security and privacy. For example, a community-wide open competition for developing novel genomic data protection methods has shown the feasibility of secure data outsourcing and collaboration for cloud-based genomic data analysis [61]. The findings from the work demonstrate that cryptographic techniques can support public cloud-based comparative analysis of human genomes. Recent work has shown that by using a hybrid cloud deployment model, 50%–70% of the read mapping task can be carried out accurately and efficiently in a public cloud [62].

In summary, a cloud-based ecosystem requires capability for interoperability between clouds, development of tools that can operate in multiple cloud environments and that can address the challenges of data protection, privacy, and legal constraints imposed by different countries (see [63] for a discussion as it relates to genomic data).

## Cloud advantages and disadvantages for biomedical projects

Cloud costs vary among biomedical projects and among vendors, so defining technical requirements for provisioning resources (e.g., amount of memory, disk storage, and CPU use) is an important first step in estimating costs. Remember the intent of commercial public cloud providers is to have you continue to use their cloud environment. For example, data may be free to upload but expensive to download, making adoption of commons approach in the cloud even more important for hosting large-scale biological data sets. This approach can meet community needs of data producers, consumers, and stewards (Fig 1) to improve access and minimize the need for downloading sets to local institutions. To test this approach, NIH has initiated a data commons pilot [64] by supporting the hosting of 3 important data sets, namely, Trans-Omics Precision Medicine initiative (TOPMed), Genotype Tissue Expression project (GTEx), and Alliance of Genomics Resource link, a consortium for Model Organism Databases (MODS) in the cloud.

Many cloud providers make available calculators for estimating approximate usage costs for their respective cloud services [65]. Without any point of reference to start with, estimating costs may be challenging. Commercial public cloud providers generally offer free credit with new accounts, which may be sufficient to kickstart the planning and evaluation process. Cloud service charges are based on exact usage in small time increments, whereas on site compute costs are typically amortized over 3–5-year periods for systems that can be used for multiple projects. Though cost comparisons between local infrastructure and cloud approaches are frequently sought, in practice, such comparisons are often difficult to perform effectively due to the lack of good data for actual local costs. Moreover, funding models for cloud computing differ among institutions receiving the funds and the funders themselves. For example, use of cloud resources may be subject to institutional overhead, whereas on-site hardware may not. This is not the best use of taxpayer money, and funding agencies should review their policies with respect to cloud usage by institutions charging overhead. Given the growing competitiveness in the cloud market, cloud resources may be negotiable or available under special agreements for qualifying research and education projects [66–68].

Biomedical researchers in collaboration with IT professionals will need to determine the best way to leverage cloud resources for their individual projects [69]. Computing costs for using on premise infrastructure requires determining the total cost of ownership (TCO). Both direct and indirect costs contribute towards TCO. Direct costs include hardware purchase costs, network services, data center, electricity, software licenses, and salaries. Indirect costs typically include technical support services, data management, and training. Indirect institutional costs vary significantly depending on the complexity of the project. Productivity is a consideration when assessing costs. For example, a whole genome pipeline in a cloud environment, once prototyped, can be scaled up for processing entire genomes with subsequent minimal human cost [70].

Using idle computing nodes in the cloud that are preemptible is one of the ways to reduce computing cost, but at the risk of increasing time to compute. For example, a recent report using the NCI cloud pilot ISB-CGC for quantification of transcript-expression levels for over 12,000 RNA-sequencing samples on 2 different cloud-based configurations, cluster-based versus pre-emptible, showed that the per sample cost when using the pre-emptible configuration was less than half the cost compared to the cluster-based method [71].

Other approaches have used linear programming methods to optimally bid on and deploy a combination of underutilized computing resources in genomics to minimize the cost of data analysis [72].

Cloud environments are pay-as-you-go, whereas research funding for computation is typically given at the beginning of an award and estimated on an annual basis. This can lead to a mismatch between the need for compute and the resources to meet that need. The NIH undertook a cloud credits pilot to assess an alternative funding model for cloud resources, details will be fully described in [73]. Credits were awarded when needed as opposed to up front, thereby matching usage patterns. A simple application and review mechanism available to a funded investigator means credits can typically be awarded in weeks or less. The investigator can choose with which cloud provider to spend the credits, thereby driving competition into the marketplace and presumably increasing the amount of compute that can be performed on research monies.

Cloud credits have focused on incentivizing cloud usage; however, a challenge that remains to be addressed is longer term data sustainability in cloud environments. The cost for data management and storage for retaining all the data produced during a research program can be prohibitive as collections become large. One of the ways to proactively tackle this issue is by engaging data producers, consumers, and curators from the beginning of the research data lifecycle process for developing value-based models for data retention, which can be implemented via cloud storage. Based on usage patterns, a policy driven data migration to least expensive cloud storage pools can be adopted. Our perspective is that long-term retention of biomedical data is an excellent venue for public and private institutions to partner together, to explore ways for co-ownership to manage cost and policy that can continue to make research data accessible over time.

## Summary and conclusions

Cloud usage, from large-scale genomics analysis to remote monitoring of patients to molecular diagnostics work in clinical laboratories, has advantages but also potential drawbacks. A first step is the determination of what type of cloud environment best fits the application and then whether it represents a cost-effective solution. This introduction attempts to indicate what should be considered, what the options are, and what applications are already in use that may serve as references in making the best determination on how to proceed.

Cloud vendors provide multiple services for compute, storage, deployment of virtual machines, and access to various databases. Cloud vendors and third parties provide additional services to map users ranging from novices to experts. The ubiquitous nature of clouds raises questions regarding security and accessibility, particularly as it relates to geopolitical boundaries. Cost benefits of using clouds over other compute environments need to be carefully assessed as they relate to the size, complexity, and nature of the task. Clouds are termed elastic as they expand to embrace the compute needs of a task. For example, a simple, small prototype can be tested in a cloud environment and immediately scaled up to handle very large data. On the other hand, there is a cost associated with such usage, particularly in extricating the outcomes of the computation. Cloud vendors are seeking an all-in model. Once you commit to using their services, you continue to do so or pay a significant penalty. This, combined with being a pay-as-you-go model, has implications when mapped to the up-front funding models of typical grants. The idea of environments where multiple public cloud providers are used in a collective ecosystem is still mostly on the horizon. What is clear, however, is that clouds are a growing part of the biomedical computational ecosystem and are here to stay.

## References

[1] FedRAMP.gov. In: FedRAMP.gov [Internet]. [cited 18 Sep 2017]. Available from: https://www.fedramp.gov/

[2] Indiana University Pervasive Technology Institute. Jetstream: A National Science and Engineering Cloud [Internet]. [cited 19 Sep 2017]. Available from: https://jetstream-cloud.org/

[3] Mell P, Grance T, Others. The NIST definition of cloud computing [Internet]. [cited 18 Sep 2017]. National Institute of Standards and Technology; 2011. Report No.: Special Publication 800–145. Available from: http://nvlpubs.nist.gov/nistpubs/Legacy/SP/nistspecialpublication800-145.pdf

[4] Palian J. Cloud Computing Adoption Across Industries. In: Expedient [Internet]. 19 Mar 2013 [cited 18 Sep 2017]. Available from: https://www.expedient.com/blog/how-cloud-computing-adoption-varies-across-industries/

[5] Amazon Web Services. AWS Public Datasets. In: AWS Public Datasets [Internet]. [cited 18 Sep 2017]. Available from: https://aws.amazon.com/datasets/

[6] Amazon Web Services. Human Microbiome Project on Amazon Web Services. In: Amazon Web Services Public Datasets [Internet]. [cited 18 Sep 2017]. Available from: https://aws.amazon.com/datasets/human-microbiome-project/

[7] National Institutes of Health. Use of Cloud Computing Services for Storage and Analysis of Controlled-Access Data Subject to the NIH Genomic Data Sharing Policy [Internet]. [cited 18 Sep 2017]. Available from: https://gds.nih.gov/pdf/NIH_Position_Statement_on_Cloud_Computing.pdf

[8] National Institutes of Health. National Institutes of Health Plan for Increasing Access to Scientific Publications and Digital Scientific Data from NIH Funded Scientific Research [Internet]. [cited 18 Sep 2017]. NIH; 2015 Feb. Available from: https://grants.nih.gov/grants/NIH-Public-Access-Plan.pdf

[9] Dai L, Gao X, Guo Y, Xiao J, Zhang Z. Bioinformatics clouds for big data manipulation. Biol Direct. BioMed Central; 2012;7: 43. 10.1186/1745-6150-7-43 PMC3533974 23190475

[10] Camacho C, Coulouris G, Avagyan V, Ma N, Papadopoulos J, Bealer K, et al BLAST+: architecture and applications. BMC Bioinformatics. 2009;10: 421 doi: 10.1186/1471-2105-10-421 20003500 10.1186/1471-2105-10-421 PMC2803857

[11] NCBI. Cloud BLAST. In: Cloud BLAST Documentation [Internet]. [cited 18 Sep 2017]. Available from: https://blast.ncbi.nlm.nih.gov/Blast.cgi?PAGE_TYPE=BlastDocs&DOC_TYPE=CloudBlast

[12] NCBI BLAST on Windows Azure. In: Microsoft Download Center [Internet]. [cited 18 Sep 2017]. Available from: https://www.microsoft.com/en-us/download/details.aspx?id=52513

[13] Nguyen T, Shi W, Ruden D. CloudAligner: A fast and full-featured MapReduce based tool for sequence mapping. BMC Res Notes. 2011;4: 171 doi: 10.1186/1756-0500-4-171 21645377 10.1186/1756-0500-4-171 PMC3127959

[14] Schatz MC. CloudBurst: highly sensitive read mapping with MapReduce. Bioinformatics. Oxford University Press; 2009;25: 1363–1369. doi: 10.1093/bioinformatics/btp236 19357099 10.1093/bioinformatics/btp236 PMC2682523

[15] Jourdren L, Bernard M, Dillies M-A, Le Crom S. Eoulsan: a cloud computing-based framework facilitating high throughput sequencing analyses. Bioinformatics. 2012;28: 1542–1543. doi: 10.1093/bioinformatics/bts165 22492314 10.1093/bioinformatics/bts165

[16] Langmead B, Schatz MC, Lin J, Pop M, Salzberg SL. Searching for SNPs with cloud computing. Genome Biol. 2009;10: R134 doi: 10.1186/gb-2009-10-11-r134 19930550 10.1186/gb-2009-10-11-r134 PMC3091327

[17] Afgan E, Baker D, Van den Beek M, Blankenberg D, Bouvier D, Čech M, et al The Galaxy platform for accessible, reproducible and collaborative biomedical analyses: 2016 update. Nucleic Acids Res. Oxford University Press; 2016;44: W3–W10. doi: 10.1093/nar/gkw343 27137889 10.1093/nar/gkw343 PMC4987906

[18] Taylor J. Galaxy on the Cloud. In: Coursera [Internet]. [cited 18 Sep 2017]. Available from: https://www.coursera.org/learn/galaxy-project/lecture/veQKq/galaxy-on-the-cloud

[19] Liu B, Madduri RK, Sotomayor B, Chard K, Lacinski L, Dave UJ, et al Cloud-based bioinformatics workflow platform for large-scale next-generation sequencing analyses. J Biomed Inform. 2014;49: 119–133. doi: 10.1016/j.jbi.2014.01.005 24462600 10.1016/j.jbi.2014.01.005 PMC4203338

[20] Heath AP, Greenway M, Powell R, Spring J, Suarez R, Hanley D, et al Bionimbus: a cloud for managing, analyzing and sharing large genomics datasets. J Am Med Inform Assoc. 2014;21: 969–975. doi: 10.1136/amiajnl-2013-002155 24464852 10.1136/amiajnl-2013-002155 PMC4215034

[21] Home—OpenStack Open Source Cloud Computing Software. In: OpenStack [Internet]. [cited 16 Oct 2017]. Available from: https://www.openstack.org/

[22] McNerney ME, Brown CD, Wang X, Bartom ET, Karmakar S, Bandlamudi C, et al CUX1 is a haploinsufficient tumor suppressor gene on chromosome 7 frequently inactivated in acute myeloid leukemia. Blood. 2013;121: 975–983. doi: 10.1182/blood-2012-04-426965 23212519 10.1182/blood-2012-04-426965 PMC3567344

[23] Afgan E, Baker D, Coraor N, Chapman B, Nekrutenko A, Taylor J. Galaxy CloudMan: delivering cloud compute clusters. BMC Bioinformatics. 2010;11 Suppl 12: S4. 10.1186/1471-2105-11-S12-S4 PMC3040530 21210983

[24] Onsongo G, Erdmann J, Spears MD, Chilton J, Beckman KB, Hauge A, et al Implementation of Cloud based Next Generation Sequencing data analysis in a clinical laboratory. BMC Res Notes. 2014;7: 314 doi: 10.1186/1756-0500-7-314 24885806 10.1186/1756-0500-7-314 PMC4036707

[25] PanCancer Analysis Working Group. In: ICGC Data Portal [Internet]. [cited 19 Sep 2017]. Available from: https://dcc.icgc.org/pcawg

[26] International Cancer Genome Consortium. Cancer Collaboratory. In: Cancer Collaboratory [Internet]. [cited 19 Sep 2017]. Available from: https://dcc.icgc.org/icgc-in-the-cloud/collaboratory

[27] Stein LD, Knoppers BM, Campbell P, Getz G, Korbel JO. Data analysis: Create a cloud commons. Nature. 2015;523: 149–151. doi: 10.1038/523149a 26156357 10.1038/523149a

[28] National Cancer Institute. National Cancer Institute Cancer Genomics Cloud Pilots [Internet]. [cited 18 Sep 2017]. Available from: https://cbiit.cancer.gov/sites/nci-cbiit/files/Cloud_Pilot_Handout_508compliant.pdf

[29] Grossman RL, Heath AP, Ferretti V, Varmus HE, Lowy DR, Kibbe WA, et al Toward a Shared Vision for Cancer Genomic Data. N Engl J Med. 2016;375: 1109–1112. doi: 10.1056/NEJMp1607591 27653561 10.1056/NEJMp1607591 PMC6309165

[30] Broad Institute. FIRECLOUD. In: FireCloud [Internet]. [cited 18 Sep 2017]. Available from: https://software.broadinstitute.org/firecloud/

[31] Broad Institute. Workflow Description Language [Internet]. [cited 19 Sep 2017]. Available from: https://software.broadinstitute.org/wdl/

[32] Institute for Systems Biology. Institute for Systems Biology: Cancer Genomics Cloud [Internet]. [cited 19 Sep 2017]. Available from: http://cgc.systemsbiology.net/

[33] SevenBridges Genomics. Cancer Genomics Cloud. In: Cancer Genomics Cloud [Internet]. [cited 19 Sep 2017]. Available from: http://www.cancergenomicscloud.org/

[34] Amstutz P, Crusoe MR, Tijanić N, Chapman B, Chilton J, Heuer M, et al. Common Workflow Language, v1.0. figshare. 2016; doi: 10.6084/m9.figshare.3115156.v2

[35] Sinha S, Song J, Weinshilboum R, Jongeneel V, Han J. KnowEnG: a knowledge engine for genomics. J Am Med Inform Assoc. American Medical Informatics Association; 2015;22: 1115 doi: 10.1093/jamia/ocv090 26205246 10.1093/jamia/ocv090 PMC5009907

[36] Anderson C. AZ Partners with DNAnexus for 2 Million Patient Sequencing Project. Clinical OMICs. Mary Ann Liebert, Inc., publishers; 2017;4: 32–32.

[37] DNAnexus Platform Offers Google-Developed DeepVariant | GEN. In: GEN [Internet]. 13 Dec 2017 [cited 26 Jan 2018]. Available from: https://www.genengnews.com/gen-news-highlights/dnanexus-platform-offers-google-developed-deepvariant/81255267

[38] DNAstack—Genomics made simple [Internet]. [cited 28 Jan 2018]. Available from: https://dnastack.com/#/team/mission

[39] Pandey S, Voorsluys W, Niu S, Khandoker A, Buyya R. An autonomic cloud environment for hosting ECG data analysis services. Future Gener Comput Syst. 2012;28: 147–154.

[40] Hsieh J-C, Hsu M-W. A cloud computing based 12-lead ECG telemedicine service. BMC Med Inform Decis Mak. 2012;12: 77 doi: 10.1186/1472-6947-12-77 22838382 10.1186/1472-6947-12-77 PMC3461479

[41] Silva LAB, Costa C, Oliveira JL. A PACS archive architecture supported on cloud services. Int J Comput Assist Radiol Surg. 2012;7: 349–358. doi: 10.1007/s11548-011-0625-x 21678039 10.1007/s11548-011-0625-x

[42] Griebel L, Prokosch H-U, Köpcke F, Toddenroth D, Christoph J, Leb I, et al A scoping review of cloud computing in healthcare. BMC Med Inform Decis Mak. 2015;15: 17 doi: 10.1186/s12911-015-0145-7 25888747 10.1186/s12911-015-0145-7 PMC4372226

[43] Angiuoli SV, Matalka M, Gussman A, Galens K, Vangala M, Riley DR, et al CloVR: a virtual machine for automated and portable sequence analysis from the desktop using cloud computing. BMC Bioinformatics. 2011;12: 356 doi: 10.1186/1471-2105-12-356 21878105 10.1186/1471-2105-12-356 PMC3228541

[44] Krampis K, Booth T, Chapman B, Tiwari B, Bicak M, Field D, et al Cloud BioLinux: pre-configured and on-demand bioinformatics computing for the genomics community. BMC Bioinformatics. 2012;13: 42 doi: 10.1186/1471-2105-13-42 22429538 10.1186/1471-2105-13-42 PMC3372431

[45] Google. An Inside Look at Google BigQuery [Internet]. [cited 19 Sep 2017]. Google; 2012. Available from: https://cloud.google.com/files/BigQueryTechnicalWP.pdf

[46] AWS. Enterprise Data Warehousing on Amazon Web Services [Internet]. [cited 19 Sep 2017]. 2016. Available from: https://d0.awsstatic.com/whitepapers/enterprise-data-warehousing-on-aws.pdf

[47] Luo J, Wu M, Gopukumar D, Zhao Y. Big Data Application in Biomedical Research and Health Care: A Literature Review. Biomed Inform Insights. SAGE Publications; 2016;8: 1. 10.4137/BII.S31559 PMC4720168 26843812

[48] Wilkinson MD, Dumontier M, Aalbersberg IJJ, Appleton G, Axton M, Baak A, et al The FAIR Guiding Principles for scientific data management and stewardship. Sci Data. 2016;3: 160018 doi: 10.1038/sdata.2016.18 26978244 10.1038/sdata.2016.18 PMC4792175

[49] Jagodnik KM, Koplev S, Jenkins SL, Ohno-Machado L, Paten B, Schurer SC, et al Developing a framework for digital objects in the Big Data to Knowledge (BD2K) commons: Report from the Commons Framework Pilots workshop. J Biomed Inform. 2017;71: 49–57. doi: 10.1016/j.jbi.2017.05.006 28501646 10.1016/j.jbi.2017.05.006 PMC5545976

[50] Grossman RL, Heath A, Murphy M, Patterson M, Wells W. A Case for Data Commons: Toward Data Science as a Service. Comput Sci Eng. 2016;18: 10–20. doi: 10.1109/MCSE.2016.92 29033693 10.1109/MCSE.2016.92 PMC5636009

[51] Ohno-Machado L, Sansone S-A, Alter G, Fore I, Grethe J, Xu H, et al Finding useful data across multiple biomedical data repositories using DataMed. Nat Genet. 2017;49: 816–819. doi: 10.1038/ng.3864 28546571 10.1038/ng.3864 PMC6460922

[52] Vukicevic M, Radovanovic S, Milovanovic M, Minovic M. Cloud Based Metalearning System for Predictive Modeling of Biomedical Data. The Scientific World Journal. Hindawi; 2014;2014 doi: 10.1155/2014/859279 24892101 10.1155/2014/859279 PMC4032768

[53] Szczerba M, Wiewiórka MS, Okoniewski MJ, Rybiński H. Scalable Cloud-Based Data Analysis Software Systems for Big Data from Next Generation Sequencing Big Data Analysis: New Algorithms for a New Society. Springer, Cham; 2016 pp. 263–283.

[54] Foster I, Gannon DB. Cloud Computing for Science and Engineering. MIT Press; 2017.

[55] Data4Cure I. Data4Cure :: Biomedical Intelligence [Internet]. [cited 2 Feb 2018]. Available from: https://www.data4cure.com/solutions.html

[56] O’Reilly PD. Federal Information Security Management Act (FISMA) Implementation Project. 2009; [internet]. [cited 7 Feb 2018]. Available from:https://www.nist.gov/programs-projects/federal-information-security-management-act-fisma-implementation-project

[57] Ross RS. Security and Privacy Controls for Federal Information Systems and Organizations [includes updates as of 5/7/13] [Internet].[cited 7 Feb 2018]. 2013. Available from: https://www.nist.gov/publications/security-and-privacy-controls-federal-information-systems-and-organizations-includes

[58] Author: National Institute of Standards, Technology. FIPS 200, Minimum Security Requirements for Federal Info and Info Systems | CSRC [Internet]. [cited 7 Feb 2018]. Available from: http://csrc.nist.gov/publications/fips/fips200/

[59] Malin BA, Emam KE, O’Keefe CM. Biomedical data privacy: problems, perspectives, and recent advances. J Am Med Inform Assoc. 2013;20: 2–6. doi: 10.1136/amiajnl-2012-001509 23221359 10.1136/amiajnl-2012-001509 PMC3555341

[60] Schaefer T, CISA, CISM, CISSP, CEH, Hofmann M, et al Selecting the Right Cloud Operating Model: Privacy and Data Security in the Cloud. ISACA. 2014;3 [Internet]. [cited 7 Feb 2018]. Available from: https://www.isaca.org/Journal/archives/2014/Volume-3/Pages/Selecting-the-Right-Cloud-Operating-Model-Privacy-and-Data-Security-in-the-Cloud.aspx

[61] Tang H, Jiang X, Wang X, Wang S, Sofia H, Fox D, et al Protecting genomic data analytics in the cloud: state of the art and opportunities. BMC Med Genomics. 2016;9: 63 doi: 10.1186/s12920-016-0224-3 27733153 10.1186/s12920-016-0224-3 PMC5062944

[62] Popic V, Batzoglou S. A hybrid cloud read aligner based on MinHash and kmer voting that preserves privacy. Nat Commun. 2017;8: 15311 doi: 10.1038/ncomms15311 28508884 10.1038/ncomms15311 PMC5440850

[63] Molnár-Gábor F, Lueck R, Yakneen S, Korbel JO. Computing patient data in the cloud: practical and legal considerations for genetics and genomics research in Europe and internationally. Genome Med. 2017;9: 58 doi: 10.1186/s13073-017-0449-6 28633659 10.1186/s13073-017-0449-6 PMC5477758

[64] Data Science at NIH [Internet]. [cited 1 Feb 2018]. Available from: https://datascience.nih.gov/DataCommonsPilotPhaseAwards

[65] Google Cloud Platform Pricing Calculator -. In: Google Cloud Platform [Internet]. [cited 22 Sep 2017]. Available from: https://cloud.google.com/products/calculator/

[66] Amazon Web Services. AWS Programs for Research and Education. In: AWS Programs for Research and Education [Internet]. [cited 22 Sep 2017]. Available from: https://aws.amazon.com/grants/

[67] Google. Education Grants—Free Credits for University Computer Science Classes | Google Cloud Platform. In: Google Cloud Platform [Internet]. [cited 22 Sep 2017]. Available from: https://cloud.google.com/edu/

[68] Microsoft. Microsoft Azure for Research—Microsoft Research. In: Microsoft Research [Internet]. [cited 22 Sep 2017]. Available from: https://www.microsoft.com/en-us/research/academic-program/microsoft-azure-for-research/

[69] Knaus J, Hieke S, Binder H, Schwarzer G, Others. Costs of Cloud Computing for a Biometry Department. Methods Inf Med. Schattauer Publishers; 2013;52: 72–79. doi: 10.3414/ME11-02-0048 23188548 10.3414/ME11-02-0048

[70] Fusaro VA, Patil P, Gafni E, Wall DP, Tonellato PJ. Biomedical cloud computing with Amazon Web Services. PLoS Comput Biol. 2011;7: e1002147 doi: 10.1371/journal.pcbi.1002147 21901085 10.1371/journal.pcbi.1002147 PMC3161908

[71] Tatlow PJ, Piccolo SR. A cloud-based workflow to quantify transcript-expression levels in public cancer compendia. Sci Rep. 2016;6: 39259 doi: 10.1038/srep39259 27982081 10.1038/srep39259 PMC5159871

[72] Luber JM, Tierney BT, Cofer EM, Patel CJ, Kostic AD. Aether: Leveraging Linear Programming For Optimal Cloud Computing In Genomics. Bioinformatics. 2017; doi: 10.1093/bioinformatics/btx787 29228186 10.1093/bioinformatics/btx787 PMC5925767

[73] Komatsoulis GA, Weber N, Tenenbaum D, Bourne PE. The Commons Credit Model: A New Funding Approach with Potential to Improve Computational Biomedicine. PLoS Biol. Submitted.

